# Dicentrine Potentiates TNF-α-Induced Apoptosis and Suppresses Invasion of A549 Lung Adenocarcinoma Cells via Modulation of NF-κB and AP-1 Activation

**DOI:** 10.3390/molecules24224100

**Published:** 2019-11-13

**Authors:** Chanatip Ooppachai, Pornngarm Limtrakul (Dejkriengkraikul), Supachai Yodkeeree

**Affiliations:** 1 Department of Biochemistry, Faculty of Medicine, Chiang Mai University, Chiang Mai 50200, Thailand; chanatip.fang@gmail.com (C.O.); ); 2 Anticarcinogenesis and Apoptosis Research Cluster, Faculty of Medicine, Chiang Mai University, Chiang Mai 50200, Thailand; 3 Center for Research and Development of Natural Products for Health, Chiang Mai University, Chiang Mai 50200, Thailand

**Keywords:** TNF-α, dicentrine, apoptosis, metastasis, lung adenocarcinoma

## Abstract

Numerous studies have indicated that tumor necrosis factor-alpha (TNF-α) could induce cancer cell survival and metastasis via activation of transcriptional activity of NF-κB and AP-1. Therefore, the inhibition of TNF-α-induced NF-κB and AP-1 activity has been considered in the search for drugs that could effectively treat cancer. Dicentrine, an aporphinic alkaloid, exerts anti-inflammatory and anticancer activities. Therefore, we investigated the effects of dicentrine on TNF-α-induced tumor progression in A549 lung adenocarcinoma cells. Our results demonstrated that dicentrine effectively sensitizes TNF-α-induced apoptosis in A549 cells when compared with dicentrine alone. In addition, dicentrine increases caspase-8, -9, -3, and poly (ADP-ribose) polymerase (PARP) activities by upregulating the death-inducing signaling complex and by inhibiting the expression of antiapoptotic proteins including cIAP2, cFLIP, and Bcl-XL. Furthermore, dicentrine inhibits the TNF-α-induced A549 cells invasion and migration. This inhibition is correlated with the suppression of invasive proteins in the presence of dicentrine. Moreover, dicentrine significantly blockes TNF-α-activated TAK1, p38, JNK, and Akt, leading to reduced levels of the transcriptional activity of NF-κB and AP-1. Taken together, our results suggest that dicentrine could enhance TNF-α-induced A549 cell death by inducing apoptosis and reducing cell invasion due to, at least in part, the suppression of TAK-1, MAPK, Akt, AP-1, and NF-κB signaling pathways.

## 1. Introduction

Lung cancer is one of the leading causes of cancer-associated mortality worldwide. Nonsmall-cell lung cancer (NSCLC) is responsible for more than 80% of all lung cancers. Despite the fact that advancements have been made in cancer therapies, the overall survival rate of patients has remained unchanged in recent years [1]. Several studies have provided evidence to support the hypothesis that the tissue damage caused by inflammation can initiate or promote the development of lung cancer [2,3]. Chronic inflammation has emerged as a key contributor of cancer cell survival, angiogenesis, and metastasis. Inflammatory cytokines, such as interleukin-6 (IL-6), IL-1, and tumor necrosis factor-alpha (TNF-α), can promote cancer progression [4,5,6,7]. Among them, TNF-α contributes to the survival and metastasis of lung cancer, while the level of TNF-α in the tumor tissues and serum obtained from patients with NSCLC has increased significantly along with the clinical stage of the tumor [8,9].

Accordingly, even more complicated roles for TNF-α in cancer cases have emerged. The anticancer property of TNF-α is mainly achieved by inducing cancer cell death. This pathway is initiated by TNFR1 internally signaling to form complex II, which consists of TRADD, RIP1, FADD, and caspase-8 [10,11,12]. Caspase-8 is autoactivated, leading to the initiation of the caspase cascade and the induction of apoptotic cell death [13,14]. On the other hand, TNF-α stimulates proliferation, survival, angiogenesis, and metastasis in most cancer cells that are resistant to TNF-α-induced cell death [15]. After binding to TNFR1, TNF-α can increase the expression of antiapoptotic (cIAPs, XIAP, Bcl-2, Bcl-xl, and cFLIP), angiogenic (VEGF and COX-2), and invasive (MMP-9, MT1-MMP, uPA, urokinase-type plasminogen activator receptor (uPAR), and intercellular adhesion molecule 1 (ICAM-1)) proteins via the TAK-1, MAPKs, Akt, IKK, AP-1, and NF-κB signaling pathways [10,16,17]. Therefore, the identification of sensitizing agents that are capable of suppressing TNF-α-induced survival signaling could be an attractive discovery in facilitating the enhancement of TNF-α-mediated apoptosis and tumor progression.

Dicentrine is an aporphine alkaloid found in the roots of *L. megaphylla* and several other plants [18]. Previous investigations have shown that dicentrine possesses multiple pharmacological activities, including platelet aggregation inhibition capabilities, antinociceptive and anticancer activities [19,20,21]. Recently, our previous findings have demonstrated that dicentrine inhibited the inflammation in lipopolysaccharide (LPS)-treated RAW 264.7 cells via the suppression of the AP-1, NF-κB, and MAPKs signaling pathways [22]. However, the effect of dicentrine on TNF-α-induced apoptosis and metastasis in lung cancer cells has not yet been elucidated.

In the current study, we have investigated the mechanism, by which dicentrine inhibits the TNF-α-induced expression and the survival of metastasis proteins. We have also determined the effects of dicentrine on the MAPKs, Akt, NF-κB, and AP-1 signaling pathways in TNF-α-induced A549 cells.

## 2. Results

### 2.1. Dicentrine Potentiates TNF-α-Induced Apoptosis in A549 Lung Adenocarcinoma Cells

**Figure 1:**
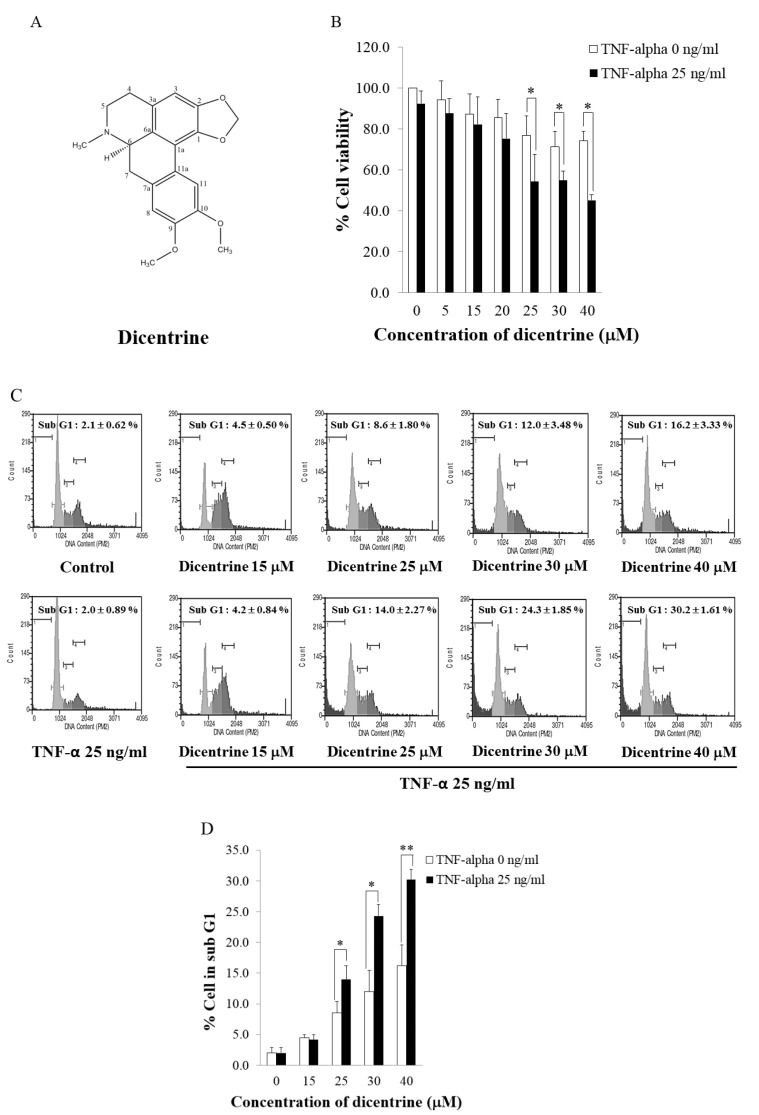
Dicentrine enhances tumor necrosis factor-alpha (TNF-α)-induced apoptosis in A549 cells. (**A**) Structure of dicentrine. A549 cells were pretreated with various concentrations of dicentrine for 4 h and then cotreated with 25 ng/mL of TNF-α for 24 h. (**B**) Cell viability was determined by 3-(4,5-dimethylthiazol-2-yl)-2,5-diphenyltetrazolium bromide (MTT) assays. (**C**,**D**) Cell cycle distribution was stained with propidium iodide (PI) and analyzed by flow cytometry to measure a SubG1 cell population, which represented the apoptotic cells. The experiments were performed in triplicate. The data are represented as mean ± S.D. * indicates *p* < 0.05, and ** indicates *p* < 0.01, compared with those treated with dicentrine alone.

To examine whether dicentrine enhanced TNF-α-induced cell death, A549 cells were incubated with dicentrine (0–40 μM) and cotreated with or without TNF-α (25 ng/mL) for 24 h. The cell viability was then determined by 3-(4,5-dimethylthiazol-2-yl)-2,5-diphenyltetrazolium bromide (MTT) assays. As is shown in Figure 1B, a treatment of A549 cells with dicentrine alone significantly decreased cell viability in a dose-dependent manner. However, a combined treatment of the cells with TNF-α and dicentrine at 25, 30, and 40 μM reduced the degrees of cell viability to 54.85%, 54.35%, and 45.15%, respectively, which significantly increased the level of cytotoxicity to a greater degree than the treatment with dicentrine alone. Next, we investigated whether dicentrine-potentiated TNF-α-induced cell death was associated with apoptosis by using propidium iodide (PI) staining assays and detecting a SubG1 cell population by flow cytometric analysis. As is shown in Figure 1C,D, the combined treatment of the cells with TNF-α and dicentrine at 25–40 μM significantly increased the number of apoptotic cells in a dose-dependent manner, when compared with the treatment of dicentrine alone.

### 2.2. Dicentrine Enhances TNF-α-Induced Apoptosis in a Caspase-Dependent Manner and Inhibits the Expression of Antiapoptotic Proteins

**Figure 2:**
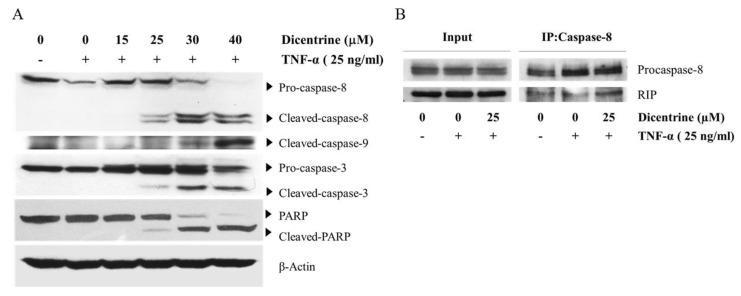

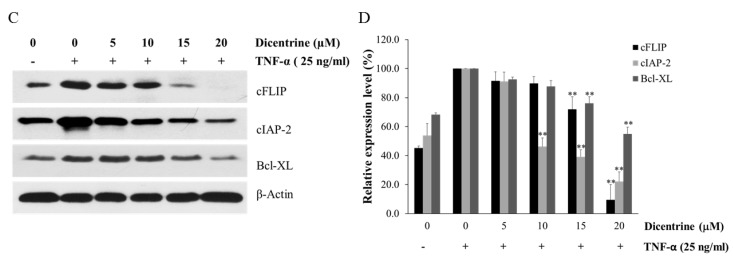
Effects of dicentrine on TNF-α-induced A549 cells apoptosis in a caspase-dependent manner and the expression of antiapoptotic proteins. A549 cells were pretreated with various concentrations of dicentrine for 4 h and then cotreated with 25 ng/mL of TNF-α to 24 h. After the combined treatment with dicentrine (0–40 μM) and TNF-α, the whole cell extracts were prepared and analyzed by the western blot analysis to detect the expression of caspase-8, -9, -3, and poly(ADP-ribose) polymerase (PARP) (**A**). The complex between procaspase-8 and receptor-interacting protein (RIP) was determined by coimmunoprecipitation after incubating with dicentrine (25 μM) for 4 h and then cotreatment with 25 ng/mL of TNF-α for 12 h (**B**). The levels of antiapoptotic proteins such as cFLIPs, cIAP-2, and Bcl-XL was measure after cotreated with dicentrine (0–20 μM) and TNF-α. (**C**,**D**). The data are represented as mean ± S.D. ** indicates *p* < 0.01, when compared to those treated with TNF-α alone.

Since apoptosis is mainly mediated by caspase enzymes, we investigated whether dicentrine affected the TNF-α-induced proteolytic processing of caspase-8, caspase-9, caspase-3, and a caspase-3 substrate poly(ADP-ribose) polymerase (PARP) cleavage using western blot analysis. Notably, the treatment of A549 cells with TNF-α alone did not induce the proteolytic processing of caspase-8, caspase-3, and PARP, when compared with the vehicle control. However, the combined treatment of TNF-α and dicentrine resulted in an increase in the cleavage of caspase-8, caspase-9, caspase-3, and PARP in a dose-dependent manner (Figure 2A). Upon the stimulation of TNF-α, RIP could interact with the FADD protein, which in turn recruited procaspase-8 to form a death-inducing signaling complex (DISC). This complex then stimulated the caspase-8 activation that subsequently induced apoptosis. Coimmunoprecipitation was performed to determine whether dicentrine enhanced TNF-α-induced apoptosis accompanied by the increased DISC formation. As shown in Figure 2B, an increased interaction between the RIP and procaspase-8 in the combined treatment with dicentrine and TNF-α was observed when compared with that in the control. Overexpression of antiapoptotic proteins, such as cIAP2, c-FLIP, and Bcl-xl, has been linked with the inhibition of TNF-α-induced apoptosis. Therefore, we examined whether dicentrine could modulate the TNF-α-induced expression of these antiapoptotic proteins. As shown in Figure 2C,D, the induction of cFLIPs, cIAP2, and Bcl-XL by TNF-α was reduced by dicentrine in a dose-dependent manner. These results showed that dicentrine effectively enhanced the apoptotic effects of TNF-α due to the downregulation of antiapoptotic proteins. 

### 2.3. Dicentrine Inhibits TNF-α-Induced A549 Cells Invasion and Migration

**Figure 3:**
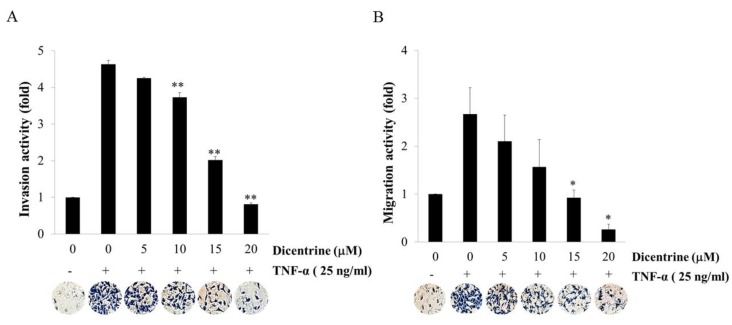
Effects of dicentrine on TNF-α-induced invasion and migration. The matrix gel was coated on membrane filters for invasion assays (**A**), and the gelatin was coated for migration assays (**B**). A549 cells with the number of 1.25 × 10^5^ cells were cultures in the upper chamber and incubated with various concentrations of dicentrine (0–20 μM) in a Dulbecco’s modified Eagle’s medium (DMEM)-free medium, and the lower chamber was filled with 25 ng/mL of TNF-α. After 24 h of incubation, the cells that actively migrated to the lower surface of the filter were determined. The data are represented as mean ± S.D. of three independent experiments. Sample groups were significantly different from the TNF-α-treated group (* indicates *p* < 0.05, and ** indicates *p* < 0.01).

Because TNF-α plays an important role in lung cancer metastasis, the effects of dicentrine on TNF-α-induced A549 cells invasion and migration were investigated. The results revealed that the invasive cells with the TNF-α treatment were increased by 4.52-fold when compared with those treated with the control, whereas dicentrine at 10–20 μM significantly decreased TNF-α-induced A549 cells invasion in a dose-dependent manner (Figure 3A). Moreover, cell migration using the treatment of the cell with TNF-α alone was increased to 2.52-fold when compared with that with the control, while the dicentrine treatment blocked the TNF-α-mediated migration in a dose-dependent manner (Figure 3B). Therefore, dicentrine may suppress the TNF-α-induced migration and the invasion of A549 cells.

### 2.4. Dicentrine Inhibits TNF-α-Induced Expression of Metastasis-Associated Proteins 

**Figure 4:**
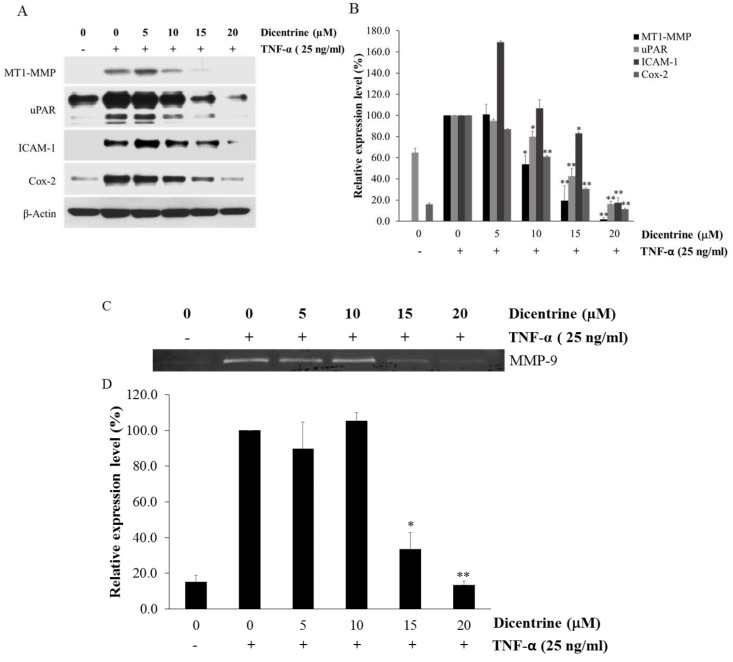
Dicentrine suppresses TNF-α-induced expression of metastatic protein. A549 cells were pretreated with various concentrations of dicentrine (0–20 μM) for 4 h and then cotreatment with 25 ng/mL of TNF-α for 24 h. The whole cell extracts were prepared and analyzed by the western blot analysis using antibodies against cell metastatic proteins (MT1-MMP, uPAR, ICAM-1, and Cox-2) (**A**,**B**). The culture supernatants of the treated cells were collected, and the secretions of MMP-9 were analyzed by gelatin zymography (**C**,**D**). The data are represented as mean ± S.D. * indicates *p* < 0.05, and ** indicates *p* < 0.01, when compared to those treated with TNF-α alone.

In the process of cancer metastasis, MT1-MMP, MMP-9, uPAR, ICAM-1, and Cox-2 are responsible for cell migration, invasion, and adhesion. The expression of these proteins was upregulated by TNF-α in various types of cancer cells including lung cancer. Therefore, the effects of dicentrine on TNF-α-induced expression of MT1-MMP, uPAR, ICAM-1, and Cox-2 in A549 cells were detected by the western blot analysis. As shown in Figure 4A,B, dicentrine at 10–20 μM significantly decreased the enhancement of TNF-α-induced MT1-MMP, uPAR, and COX-2 expression. In addition, dicentrine at 15 and 20 μM reduced the expression of ICAM-1. We next used gelatin zymography assays to examine the inhibitory effect of dicentrine on the TNF-α-induced MMP-9 secretion. As is shown in Figure 4C,D, the TNF-α-induced MMP-9 secretion was significantly inhibited in the presence of 15 and 20 μM of dicentrine.

### 2.5. Dicentrine Inhibits TNF-α-Induced NF-κB and AP-1 Activation

**Figure 5:**
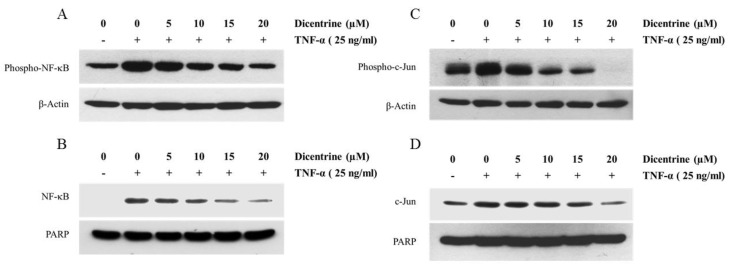
Effects of dicentrine on TNF-α-induced NF-κB and AP-1 activation. A549 cells were pretreated with difference concentrations of dicentrine (0–20 μM/mL) for 12 h and then cotreated with 25 ng/mL of TNF-α for 45 min. The phosphorylation levels of NF-κB and AP-1 in cytoplasmic extracts were detected by the western blot analysis (**A**,**C**), and the nuclear extracts were prepared to analyze the nuclear translocation (**B**,**D**). Data from a typical experiment are presented here, while similar results were obtained from three independent experiments.

The activation of NF-κB and AP-1 transcriptional activity in several types of cancer cells can promote tumor progression by regulation of many genes in terms of antiapoptosis, cell proliferation, and cell invasion. To investigate whether dicentrine affected the TNF-α-induced NF-κB and AP-1 activation, the nucleus translocation and the phosphorylation of NF-κB and AP-1 were determined. As shown in Figure 5A,B, dicentrine at 10–20 μM could significantly inhibit the TNF-α-induced p65 phosphorylation and block the TNF-α-induced nuclear translocation of p65 in a dose-dependent manner. We next tested the regulation of dicentrine on the transcription activity of AP-1. The treatment of the cells with TNF-α alone enhanced the nucleus translocation and the phosphorylation of c-Jun (AP-1). Dicentrine could significantly inhibit the TNF-α-induced AP-1 translocation and could effectively block the TNF-α-induced phosphorylation of AP-1 in a dose-dependent manner (Figure 5C,D). These results indicated that dicentrine inhibited the TNF-α-induced activation of NF-κB and AP-1 through the inhibition of the phosphorylation and the nuclear translocation of p65 and AP-1.

### 2.6. Effects of Dicentrine on TNF-α-Induced TAK-1, IκB-α, Akt, and MAPKs Signaling Pathways

**Figure 6:**
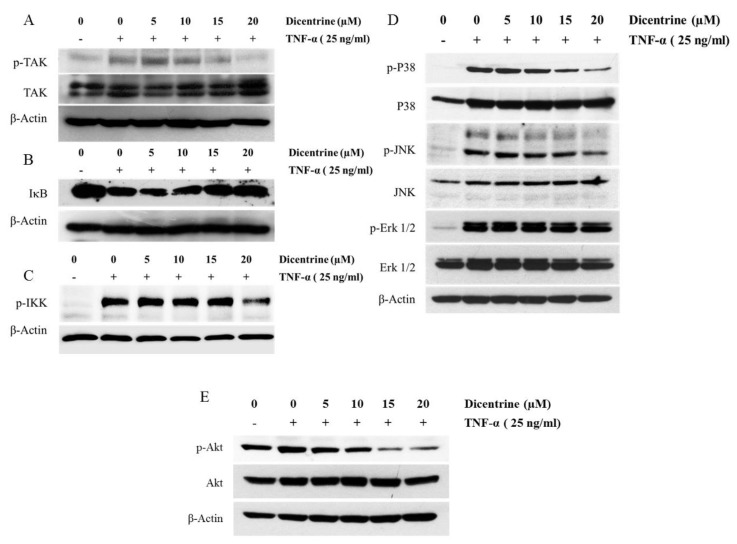
Effects of dicentrine on TNF-α-induced TAK, IKK, Akt, and MAPKs signaling pathways. A549 cells were pretreated with difference concentrations of dicentrine (0–20 μM/mL) for 12 h and then cotreated with 25 ng/mL of TNF-α for 15 min. The whole cell lysate was prepared for the measurements of phosphorylated and nonphosphorylated forms of TAK (**A**), IκB (**B**), IKK (**C**), MAPKs (**D**) and Akt (**E**) by western blot analysis. Data from a typical experiment are depicted here, and similar results were obtained in three independent experiments.

It has been reported that TNF-α-induced NF-κB and AP-1 activation is mediated through the sequential interaction of the TNF receptor with TRADD, TRAF2, and TAK-1, which then leads to the phosphorylation of IKK and induces the degradation of IκB-α, along with the phosphorylation of MAPKs and the PI3K/Akt signaling pathway. Therefore, the effects of dicentrine on the TNF-α-induced activation of TAK-1, IKK, IκB-α, Akt, and MAPKs, including Erk1/2, p38, and JNK, were investigated by the western blot analysis. As shown in Figure 6A, the treatment of A549 cells with the TNF-α-induced phosphorylation of TAK-1 and dicentrine inhibited the activation in a dose-dependent manner. The NF-κB activation by TNF-α was mediated via the IKK signaling pathway, resulting in the IκB-α degradation. To examine the effects of dicentrine on IκB-α activity, we determined whether dicentrine affected the TNF-α-induced IκB-α degradation. As is shown in Figure 6B, dicentrine blocked the TNF-α-dependent IκB-α degradation. Since the IKK complex acts as a convergence point for a variety of activating signals for NF-κB and plays a critical role in degradation of IκB-α, we investigated whether dicentrine inhibited the TNF-α-induced phosphorylation of IKK. As shown in Figure 6C, dicentrine at 20 μM suppressed the TNF-α-induced phosphorylation of IKK. Moreover, we investigated the effects of dicentrine on the TNF-α-stimulated phosphorylation of p38, JNK, and Erk1/2. As is shown in Figure 6D, dicentrine at 15 and 20 μM inhibited the TNF-α-induced phosphorylation of JNK and the p38 signaling pathway, whereas dicentrine had no effect on the TNF-α-induced phosphorylation of the ERK1/2 signaling pathway. On the other hand, the treatment with dicentrine alone also inhibited the TNF-α-induced phosphorylation of the Akt signaling pathway (Figure 6E).

## 3. Discussion

Dicentrine is an alkaloid that is found in various medicinal plants. It displays activity against many types of cancer by regulating cell cycles, inhibiting topoisomerase II, and inducing apoptosis [18,19,20,21]. Despite its various pharmacological activities, the molecular mechanism of dicentrine on TNF-α-induced tumor progression has not been adequately elucidated. The present study was designed to investigate the effect of dicentrine on the enhancement of TNF-α-induced A549 lung adenocarcinoma cell death and to investigate the role of dicentrine as a potent inhibitor of TNF-α-induced cell invasion. Molecular mechanisms of these phenomena have not yet been explored.

Activation of the TNFR1 death receptor by TNF-α can induce either cell survival or cell death, depending on the events that take place downstream of TNFR1 activation [11,17]. A recent study has shown that most cancer cells are resistant to TNF-α-induced cell death, which is known to be involved with the overexpression of antiapoptotic outcomes and the survival of proteins leading to the inhibition of the apoptotic pathway [23,24]. Therefore, the modulation of TNF-α-mediated survival signals may result in the sensitization of cancer cells to the TNF-α-induced apoptosis. Our result from the MTT assays revealed that dicentrine sensitized A549 cells to the TNF-α-induced cell death. TNF-α promotes cancer cell death by inducing apoptosis, necroptosis, and autophagy, depending on the conditions of the cells [25]. The apoptotic DNA fragmentation is being used as one of markers for apoptosis, and fractional DNA content was detected in SubG1 cells. Here, we indicated that dicentrine enhanced TNF-α-induced apoptosis by increasing the SubG1 cell population. TNF-α also induced cell apoptosis via extrinsic and intrinsic pathways by increasing the activation of caspases activity. To confirm the potential effect of dicentrine on TNF-α-mediated apoptosis in A549 cells, we investigated how dicentrine affected the TNF-α-induced activation of caspase-8, -3, -9, and PARP cleavage. The levels of caspase-8, -3, and -9 activation and the accumulation of the cleaved PARP were markedly increased during the combined treatment with TNF-α and dicentrine. During the final phase of apoptosis, caspases cleaved several proteins that are necessary for cell survival and function. Among them, PARP was cleaved by caspase-3 into the fragments. This cleavage is necessary to eliminate PARP activation in response to DNA fragmentation and prevent futile attempts of DNA repair [26]. Thus, PARP play a central role in apoptosis determining the cell fate. Taking the results together, it is possible to suggest that dicentrine potentiates TNF-α-induced apoptosis by activating caspases activity.

The TNF-α-induced apoptosis is initiated by the TNFR1 internalization into endosomes to form complex II or the DISC [11]. The DISC consists of TRADD, RIP-1, FADD, and procaspase-8. The unubiquitinated form of RIP1 can be dissociated from TNFR1, with or without TRADD, to interact with FADD, which in turn recruits procaspase-8 to form the DISC [16,27,28,29]. The formation of DISC triggers the processing and the activation of caspase-8, leading to the initiation of the caspase cascade and ultimately cell death [13,14]. Therefore, we investigated whether the enhancement effects of dicentrine on TNF-α-induced apoptosis were associated with the formation of DISC. The results from coimmunoprecipitation revealed that the combined treatment of A549 cells with dicentrine and TNF-α increased the complex formation of procaspase-8 and RIP-1. These outcomes indicated that dicentrine potentiated TNF-α-mediated apoptosis by enhancing the DISC formation. The active caspase-8 triggers a caspase cascade by the cleavage of caspase-3. Moreover, caspase-8 cleaves Bid into truncated Bid, which initiates the mitochondrial apoptosis pathway leading to the release of cytochrome c from the mitochondria. Then, cytochrome c is associated with procaspase-9 and Apat-1. This complex processes caspase-9 to an active form. Once activated, caspase-9 continues to cleave caspase-3 and eventually leads to apoptosis [13]. Our result demonstrated that dicentrine enhanced the TNF-α-induced activation of capase-9, which is one of key markers in the intrinsic pathway of apoptosis. Several studies have shown that the overexpression of antiapoptotic proteins including cIAP-2, cFLIPs, Bcl-2, and Bcl-XL in tumor cells has been associated with the inhibition of TNF-α-induced apoptosis [30,31,32]. Notably, cIAP-2 mediates ubiquitylation by controlling RIP-1 activity, thereby preventing TNF-α-induced cell death [33,34]. Moreover, c-FLIP has been identified as an inhibitor of TNF-α-mediated apoptosis by preventing the homodimerization of caspase-8 in the DISC [35,36]. Therefore, to explain the mechanism, by which dicentrine enhances TNF-α-induced apoptosis, we have examined the modulatory effects of dicentrine on the TNF-α-induced expression of the antiapoptotic capability. We found that dicentrine inhibited the TNF-α-induced expression of cIAP-2, cFLIP, and Bcl-XL. Together, these results suggested that dicentrine enhanced the TNF-α induced cell death, at least in part, by downregulating cIAP-2, cFLIP, and Bcl-XL, leading to the induction of the cell apoptosis in intrinsic and extrinsic pathways.

Tumor metastasis consists of multiple steps, including those involving loose cell–cell junctions, extracellular matrix (ECM) degradation, adherence to the surrounding ECM, and eventually migration through the ECM to the distance sites. The disruption of the basement membranes and the ECM proteins by proteolytic enzymes is an important step. MMPs and uPA are the key of protease in the ECM degradation. High levels of MMPs and uPA are closely linked to the promotion of cancer metastasis [8,15,37]. Moreover, uPAR is involved in lung cancer invasion and could enhance cell proliferation, angiogenesis, and migration. Adhesion molecules such as ICAM-1 are essential for the adhesion of tumor cells to endothelial cells and thus mediate tumor cell metastasis [38,39]. Our results demonstrated that dicentrine suppressed the TNF-α-induced A549 cell invasion and migration. Moreover, dicentrine reduced the degree of TNF-α-induced expression of invasive genes, including MMP-9, MT1-MMP, ICAM-1, and uPAR in A549 cells. In addition, Cox-2 overexpression plays a key role in tumorigenesis by stimulating angiogenesis, cell proliferation, and invasion. Cox-2 is a rate-limiting enzyme catalyzing the synthesis of prostaglandin E2 (PGE2), which induces MMPs and uPA expression [40,41]. Therefore, the modulation of Cox-2 activity is considered to be antitumor compounds. Here, we demonstrated that dicentrine reduced the TNF-α-induced expression of Cox-2 in a dose-dependent manner. This result is similar to that of our previous findings, which have stated that dicentrine reduced the levels of Cox-2 and PGE2 expression in LPS-induced macrophage cells [22].

NF-κB and AP-1 are important transcription factors that play an essential role in the progression process [41]. NF-κB controls the expression of the cell survival gene products *cIAP-2*, *Bcl-2*, *Bcl-xl*, and *cFLIP* and the invasive gene products *MMP-9*, *MT1-MMP*, *ICAM-1*, *uPA*, *uPAR*, and *Cox-2* [42,43]. In most resting cells, NF-κB exists in the cytosol in an inactive form that is associated with IκB-α. Once the cells are exposed to TNF-α, IκB-α is phosphorylated by IKK and subsequently degraded, leading to the phosphorylation and the nuclear translocation of NF-κB. The activated NF-κB then binds to the consensus sequences, activating the expression of the target genes [15,43]. Therefore, we investigated the inhibitory activity of dicentrine on the TNF-α-induced IκB-α degradation, the phosphorylation of IKK, and the NF-κB activity. The data presented herein indicate that dicentrine prevented the degradation of IκB-α and the phosphorylation of IKK and reduced the NF-κB activity by inhibiting the TNF-α-induced NF-κB phosphorylation and the nucleus translocation. This result is in accordance with the outcomes of previous studies, which also demonstrated that the inhibition of NF-κB activity can potentiate the TNF-α-induced cell death and inhibit the cancer cell invasion. AP-1 has been shown to play a role in regulating cancer cell proliferation and survival. AP-1 also controls the genes expression of *MMP-9*, *MT1-MMP*, *Cox-2*, *uPA*, and *uPAR*. The activity of AP-1 is regulated at an expression level and is involved in post-translation modification via phosphorylation. The activated AP-1 is translocated to the nucleus and is bound to the DNA consensus sequence. The inhibition of AP-1 activity have been shown to reduce cancer cell invasion and survival [44,45]. In this study, we investigated the activation of AP-1 by observing the phosphorylation and the translocation of c-Jun in the TNF-α-induced A549 cells. Our results found that dicentrine reduced the TNF-α-induced c-Jun phosphorylation and translocation to the nucleus of A549 cells. These results implied that dicentrine could reduce the level of survival and metastasis proteins by the inhibition of AP-1 and NF-κB activation.

Next, we investigated how dicentrine regulated the activity of NF-κB and AP-1 by examining its effects on several forms of kinase that function upstream of NF-κB and AP-1, such as MAPKs, Akt, and TAK1. Recent studies have indicated that TAK1 is essential for the TNF-α-induced activation of NF-κB via the activation of IKK activity. In addition, TAK1 has been implicated in TNF-α-induced MAPK activation [12,15,44]. Indeed, our study showed, for the first time, that the TNF-α-induced TAK-1 phosphorylation was inhibited by dicentrine. Notably, TNF-α can activate Akt and three MAPK cascades, including ERK1/2, p38, and JNK, which modulate the transcriptional activity of AP-1 and NF-κB. Accumulating evidence indicates that the Akt and MAPKs signaling pathways have been involved with cancer cell survival and metastasis by upregulating the expression of survival and invasive proteins [46]. On the other hand, it has been reported that high levels of reactive oxygen species promoted cancer cells apoptosis by activating the MAPKs signaling pathway [47]. Thus, the activation of MAPKs signaling pathway can induce either cancer cell death or cancer cell survival, depending on the condition of induction. In lung cancer cells, TNF-α-induced inflammation, survival, and metastasis via the MAPKs signaling pathway have been reported [48,49]. Therefore, these experiments were performed to determine whether dicentrine regulated TNF-α to stimulate the activities of Akt and MAPKs. Our results have shown that dicentrine prevented the phosphorylation of p38, JNK, and Akt. These results are consistent with those of previous reports that found the inhibitors of the PI3K/Akt and MAPKs signaling pathways can cause the cell death that is associated with apoptosis and can then reduce the invasion of cancer cells.

## 4. Materials and Methods

### 4.1. Materials

Dulbecco’s modified Eagle’s medium (DMEM), penicilin-streptomycin, and trypsin-ethylenediaminetetra acetic acid (EDTA) were purchased from GIBCO-BRL (Grand Island, NY, USA). Fetal bovine serum (FBS) was purchased from Hyclone (Logan, UT, USA). Gelatin, PI, and MTT were purchased from Sigma-Aldrich (St. Louis, MO, USA). Antibodies specific to caspase-3, caspase-8, caspase-9, cFILP, cIAP-2, Bcl-XL, COX-2, phospho-NF-κB, phospho-c-Jun, c-Jun, phospho-IKK, phospho-IκB, phospho-p38, p38, phospho-JNK, JNK, phospho-ERK1/2, ERK1/2, phospho-AKT, AKT, phospho-TAK, TAK, and RIP were purchased from Cell Signaling Technology Inc. (Beverly, MA, USA). NF-κB, PARP, MT1-MMP, uPAR, ICAM-1, and VEGF were purchased from Santa Cruz Biotechnology (Santa Cruz, CA, USA). Cyclin D1 was purchased from Milipore. The Can Get Signal^®^ Immunoreaction Enhancer Solution was purchased from Toyobo (Osaka, Japan). Matrigel was purchased from Becton Dickinson (Bedford, MA, USA). Dicentrine was ordered from Chengdu Biopurify Phytochemicals Ltd. (Sichuan, China).

### 4.2. Cell Culture

A549 lung adenocarcinoma cells (American Type Culture Collection (ATCC), CCL-185) were cultured in DMEM supplemented with 10% FBS, 100 U/mL of penicillin, and 100 μg/mL of streptomycin. The cell cultures were maintained in a humidified incubator with an atmosphere comprised of 95% air and 5% CO_2_ at 37 °C. For the dicentrine treatment, dicentrine was dissolved in dimethyl sulfoxide (DMSO) and diluted with the culture medium, and the final concentration of DMSO was less than 0.1% (*v*/*v*).

### 4.3. Cell Viability

The cytotoxic effect of dicrntrine to A549 lung adenocarcinoma cells were determined using MTT assays. Briefly, A549 cells with 5.5 × 10^3^ cells per well were placed into 96-well plates containing DMEM with 10% FBS for 24 h. After that, the cells were treated with various dicentrine concentrations (0–40 μM) with or without 25 ng/mL of TNF-α and incubated for 24 h. Then, 15 μL of the MTT solution (5 mg/mL) were added to each well and incubated at 37 °C for 4 h. After the incubation, the formazan crystals in each well were dissolved in 200 μL of DMSO. The absorbance was measured using a microplate reader at 570 nm with a reference wavelength of 630 nm.

### 4.4. Cell Cycle Arrest Assay

A549 cells were treated with various concentrations of dicentrine (0–40 μM) with or without 25 ng/mL of TNF-α for 24 h. After that, the cells were fixed with ice-cold 70% (*v*/*v*) ethanol for 15 min. The staining of the nuclear DNA content was conducted by adding PI (50 μg/mL), 0.05% triton X-100, and RNAse A (25 μg/mL) in PBS, followed by incubation at 37 °C for 30 min in the dark. Cells were washed with cold PBS and resuspended in 500 μL of PBS. The samples were analyzed by an FACScan flow cytometer, and the DNA content in the SubG1 was representative of the apoptotic cells.

### 4.5. Cell Invasion and Migration Assay

The invasion and migration were tested using the modified Boyden chamber assay as described previously. Briefly, polyvinylpyrrolidone-free polycarbonate filters (Millipore, Carrigtwohill, Tullagreen) with a pore size of 8 μm were coated with gelatin (0.01%, *w*/*v*) for cell migration or with Matrigel (12.5 μg/50 μL) for the invasion assays. The A549 cells with the number of 1.25 × 10^5^ were treated with various concentrations of dicentrine (0–20 μM/mL) and placed into the upper chamber for 24 h at 37 °C in 5% CO_2_. The medium in the lower chamber contained a serum-free culture medium of NIH3T3 cells with or without TNF-α (25 ng/mL). The cells that had invaded the lower surface of the membrane were fixed with methanol and stained with 1% (*w*/*v*) toluidine blue. The cells were migrated to the lower surface of the filter measure by count of the cell.

### 4.6. Gelatin Zymography

The secretions of MMP-9 from the cells were analyzed by gelatin zymography as described before. The culture supernatant of the treated cell was collected and separated by 10% polyacrylamide gels containing 0.1% (*w*/*v*) of gelatin in nonreducing conditions. After electrophoresis, gels were washed twice with 2.5% Triton X-100 for 30 min at room temperature to remove sodium dodecyl sulfate (SDS). The gel was then incubated at 37 °C and kept for 18 h in an activating buffer (50 mM Tris-HCl, 200 mM NaCl, and 10 mM CaCl_2_, pH 7.4). Gels were stained with Coomassie Brilliant Blue R (0.1%, *w*/*v*) and destained in 30% methanol with 10% acetic acid. The MMP-9 activity appeared as a clear band against a blue background

### 4.7. Coimmunoprecipitation and Western Blot

A549 cells with the number of 1 × 10^7^ were treated with 25 μM/mL of dicentrine for 4 h and 25 ng/mL of TNF-α for 12 h. The cells were lysed in a lysis buffer (150 mM NaCl, 50mM Tris-Hcl, 1% NP-40, 2 mM EDTA, and a proteases inhibitor mixture; Sigma-Aldrich) for 20 min on ice. Cell lysates were cleared by centrifugation at 4 °C for 10 min at 12,000 rpm. After that, incubations with Dynabeads Protein A (Thermo Fisher Scientific, Norway) and polyclonal antiRIP1 occurred overnight at 4 °C. The target protein and its complex were washed twice with the lysis buffer. For the western blot experiments, the proteins were separated by the SDS-PAGE electrophoresis and transferred to a nitrocellulose membrane (GE Healthcare Ltd., UK). Immunoblot analyses were performed using specific primary antibodies overnight at 4 °C. After being washed with TBS containing 0.3% (*v*/*w*) Tween-20 (TBST buffer), the membrane was incubated with a secondary antibody for 2 h. The blots were detected with chemiluminescence (Thermo Fisher Scientific Inc., MA, USA).

### 4.8. Extraction of Nuclear and Whole Cell Lysate

The whole cell lysate were used to determine the expression levels of apoptotic, antiapoptotic, invasive, and signaling proteins, such as caspase-3, caspase-8, PARP-1, cFLIP, cIAP-2, cyclin D1, Bcl-XL, MT1-MMP, uPAR, ICAM-1, Cox-2, phospho-TAK, TAK, phospho-IKK, phospho-IκB, phospho-p38, p38, phospho-JNK, JNK, phospho-ERK1/2, ERK1/2, phospho-AKT, and AKT in A549 cells. Briefly, the cells were pretreated with various concentrations of dicentrine for 4 h and cotreated with or without 25 ng/mL of TNF-α for 24 h. The treated cells were washed with ice-cold PBS and extracted by the RIPA buffer for 20 min on ice. The collected supernatant fraction by centrifugation at 12,000 rpm for 10 min, and the protein concentration was determined with the Bradford protein assay.

Nuclear extraction was used to determine the levels of NF-κB and AP-1 proteins. The treated cells were collected and washed twice with the ice-cold PBS. The cell pellet was extracted by a hypotonic buffer (10 mM HEPES, pH 7.9, 10 mM KCL, 0.1 mM EDTA, 0.1 mM EGTA, 1 mM dithiothreitol, 0.5 mM phenylmethylsulfonyl fluoride, 1 μg/mL leupeptin, and 1 μg/mL aprotinin) for 20 min on ice, after which 5 μL of 10% NP-40 was added. The tube were agitated on a vortex for 15 s and centrifuged at 12,000 rpm for 5 min. The supernatant was collected and was representative of the cytoplasm extract. The nuclear pellets were suspended in an ice-cold nuclear extraction buffer (20 mM HEPES, pH 7.9, 0.4 M NaCl, 1 mM EDTA, 1 mM EGTA, 1 mM dithiothreitol, 1 mM phenylmethylsulfonylfluoride, 2.0 μg/mL leupeptin, and 2.0 μg/mL aprotinin) for 25 min on ice and centrifuged at 12,000 rpm for 10 min. The supernatant was collected and was representative of the nuclear fraction.

To determine the expression level of protein in the whole cell lysate, cytoplasm and nuclear faction were separated by the SDS-PAGE electrophoresis and transferred to the nitrocellulose membrane by electroblotting. The membrane was blocked with 5% skim milk in TBS containing 0.3% (*v*/*v*) Tween-20 for 1 h and then incubated with primary antibodies at 4 °C overnight. The membrane was incubated with a secondary antibody for 2 h and detected by chemiluminescence.

### 4.9. Statistical Analysis

All data are presented as mean ± S.D. Statistical analysis was analyzed with SPSS Software using one-way ANOVA with Dunnett’s test. Statistical significance was determined at * *p* < 0.05 and ** *p* < 0.01.

## 5. Conclusions

In summary, our results suggested that dicentrine enhances TNF-α-induced A549 cell death by inducing apoptosis and by reducing cell invasion due to, at least in part, the suppression of the TAK-1, MAPKs, Akt, AP-1, and NF-κB signaling pathways. These results underscore the potential of dicentrine as a therapeutic agent in treatments of certain types of cancer, such as lung cancer, where TNF-α plays a major role in tumor progression.
